# Factory quality assurance of passive radiotherapy intensity modulators for electrons using kilovoltage x‐ray imaging

**DOI:** 10.1002/acm2.13943

**Published:** 2023-03-01

**Authors:** Andrew S. McGuffey, Garrett M. Pitcher, Rebecca L. Guidry, Kevin J. Erhart, Kenneth R. Hogstrom

**Affiliations:** ^1^ Department of Physics and Astronomy Louisiana State University Baton Rouge Louisiana USA; ^2^ Mary Bird Perkins Cancer Center Baton Rouge Louisiana USA; ^3^ .decimal, LLC Sanford Florida USA

**Keywords:** electron intensity modulation, electron therapy, planar x‐ray imaging, quality assurance

## Abstract

**Purpose:**

This work developed an x‐ray‐based method for performing factory quality assurance (QA) of Passive Radiotherapy Intensity Modulators for Electrons (PRIME) device fabrication. This method measures errors in position, diameter, and orientation of cylindrical island blocks on a hexagonal grid that comprises PRIME devices and the impact of such errors on the underlying intensity distribution.

**Methods:**

X‐ray images were acquired of six PRIME devices, which modeled three error cases (small random, large random, and systematic errors) for two island block diameters (0.158 and 0.352 cm). Island blocks in each device, 0.6 cm long tungsten cylinders of constant diameter, were spaced 0.6 cm on a hexagonal grid over approximately 8 cm square. Using a 50 kVp x‐ray image, each island block projected a racetrack, whose perimeter was fit to a function that allowed determination of its position, diameter, and angular orientation (*θ*, *ϕ*). These measured parameters were input into a pencil beam algorithm (PBA) dose calculation performed in water (16 MeV, SSD = 103 cm) for each device. PBA calculated intensity distributions using measured and planned (exact) island block parameters were compared.

**Results:**

*Θ* distributions for the 0.158 and 0.352 cm devices were nearly identical for each error case, with *θ* values for most island blocks being within 3.2°, 8.5°, and 7.5° for the small random, large random, and systematic error PRIME devices, respectively. Corresponding intensity differences between using measured and planned island block parameters were within 1.0% and 2.8% (small random), 2.2% and 4.8% (large random), and 3.2% and 6.7% (systematic) for the 0.158 and 0.352 cm devices, respectively.

**Conclusion:**

This approach provides a viable and economical method for factory QA of fabricated PRIME devices by determining errors in their planned intensity distribution from which their quality can be assessed prior to releasing to the customer.

## 1 INTRODUCTION

Electron radiotherapy is an effective modality for treating planning target volumes (PTVs) within 6 cm of the patient surface, for example, postmastectomy chest wall, head and neck, and extremities.^1^, ^2^, ^3^, ^4^ The range (*R_p_*) and sharp distal falloff (*R*_90‐10_) of 6–20 MeV electron beams allow sparing of distal normal tissues and critical structures.

Due to the lateral variation of the depth of the distal PTV surface, this tissue sparing has been shown to benefit from range modulation through the use of a variable thickness bolus.^5^, ^6^, ^7^, ^8^ Such bolus electron conformal therapy (BECT) has been studied for multiple sites^8^, ^9^, ^10^, ^11^, ^12^, ^13^, ^14^, ^15^, ^16^, ^17^ and is widely available for the clinic. Generally, the bolus is shaped so that the distal 90% isodose surface conforms to the distal surface of the PTV. However, irregularities in the proximal bolus surface can cause regions of increased dose (hot spots) as great as 120%, or decreased dose (cold spots) in the PTV due to perturbation of the electron scattering distribution.^11^ Kudchadker et al.,^11^ Doiron,^18^ and Hilliard et al.^19^ showed for such cases that intensity modulation followed by slight bolus modification can restore dose homogeneity to near its ideal value (90%–100%).

**FIGURE 1:**
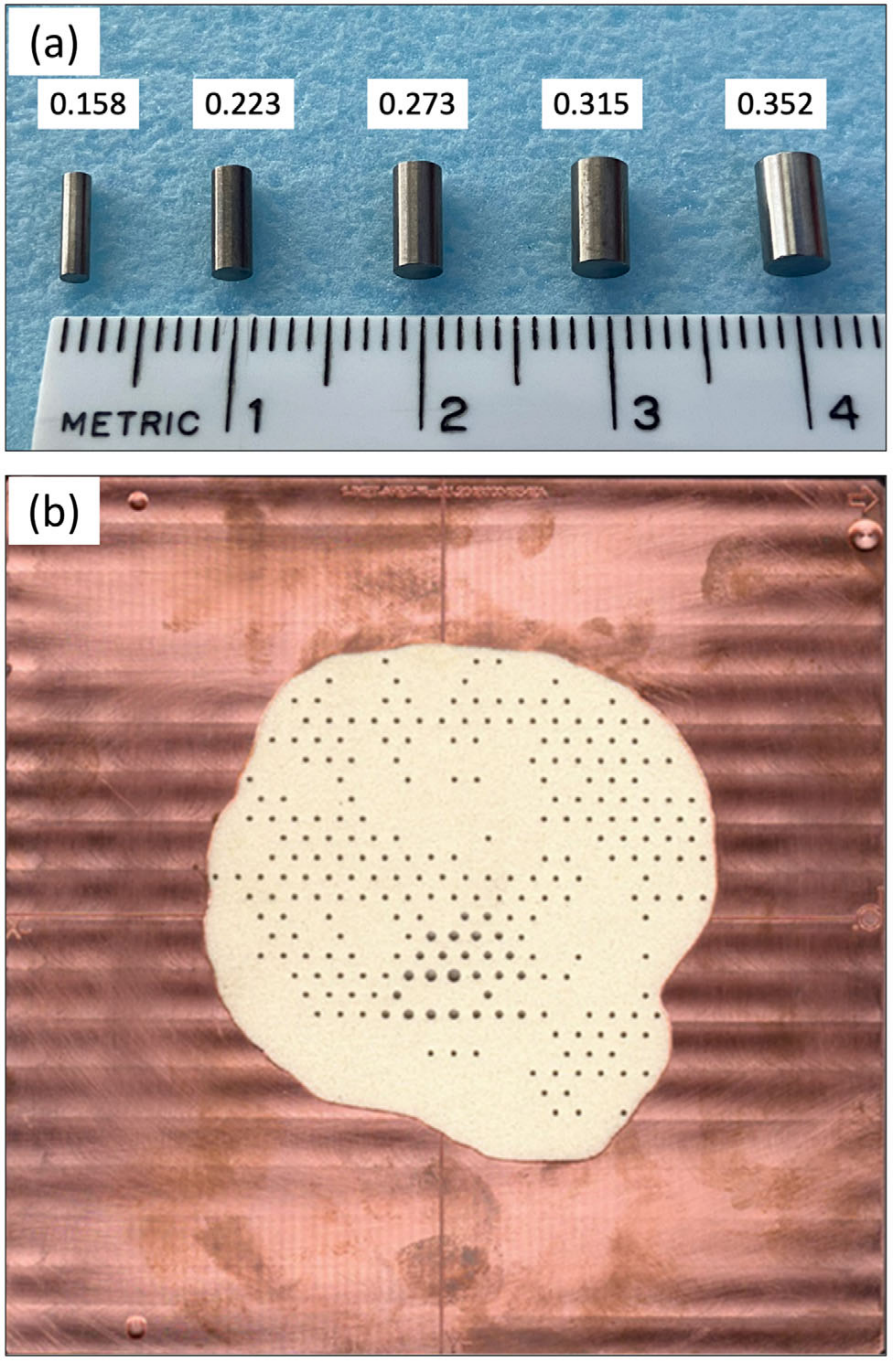
Fabricated PRIME device. (a) Island blocks are 0.6 cm long cylindrical tungsten rods that are selected from a finite set of diameters. This set has 0.158, 0.223, 0.273, 0.315, and 0.352 cm diameters having nominal IRF values of 0.937, 0.875, 0.812, 0.750, and 0.688, respectively, when placed on a hexagonal grid with 0.6 cm spacing. (b) The PRIME device fabricated based on the patient treatment plan from Hilliard et al.^19^ is shown. Island blocks of varying diameter are inserted along diverging guide holes located on a hexagonal grid (0.6 cm spacing). Diverging holes are drilled into a piece of low density, machinable foam, which fits snugly inside the patient copper cutout in a nominal 20 × 20 cm^2^ Elekta electron applicator.

To provide a practical, clinical method for electron intensity modulation delivery, Hogstrom et al. recently developed and patented the Passive Radiotherapy Intensity Modulator for Electrons (PRIME) device.^20^, ^21^ These PRIME devices, which are manufactured by .decimal, LLC (Sanford, FL, USA), consist of a matrix of 0.6 cm long cylindrical tungsten rods (island blocks) of varying diameters embedded in a low‐density, machinable foam that is placed within the patient‐specific cutout,^22^ as shown in Figure 1. The island blocks are situated on a hexagonal grid with 0.6 cm separation and oriented with their central axes projecting back to the nominal source position (specified as 100 cm upstream of isocenter on central axis), such that they align with the divergence of the beam. These blocks modulate the beam intensity, preferentially reducing fluence at specified locations across the field that correspond to regions of increased dose. The fluence is locally reduced to approximately the Intensity Reduction Factor (IRF),^21^ which is calculated as the local fractional cross‐sectional area of the beam not occupied by the island block.

In the process of IM‐BECT treatment planning and delivery, as was recently described by Hilliard et al.,^19^ patient‐specific PRIME devices were designed and ordered from .decimal, LLC, where they were manufactured and shipped to the clinic. Once received, dosimetric quality assurance (QA) was performed, using a scanning diode and water tank. To minimize the probability that a PRIME device fails dosimetric QA, it is prudent to have a factory QA process to ensure the fabricated device meets specifications.

Heretofore, factory QA of PRIME devices has been performed using optical scans of the device, which restricts evaluation of the manufactured device to the correct field shape and island block diameters and locations. However, due to the opacity of the machinable foam in which they are embedded, this method is unable to identify island blocks having correctly located upstream surfaces but erroneous insert angles such that their cylindrical axis does not follow beam divergence from the source, that is, having a misplaced downstream island block surface. Such island block misorientation was uncovered as a potential failure mode in measurements by Scotto.^23^ Therefore, this work developed a novel analysis method using kVp x‐ray imaging to perform factory QA to verify that the diameter, location, and orientation of island blocks within a PRIME device meet specification.

## 2 METHODS

Heretofore, factory QA was performed by visual inspection of optical images of the PRIME devices. Such a QA method was insufficient to detect errors in island block angle of insertion, which negatively impacted resulting underlying intensity patterns. Description of a practical, robust x‐ray imaging process is described, and results illustrating its ability to detect random and systematic errors are presented.

### 2.1 Fabrication of PRIME devices

PRIME devices were fabricated in three steps. First, a multi‐axis CNC machine drilled guide holes in a 1.27‐cm thick low density (0.096 g·cm^−2^), machinable foam board. Holes were drilled at locations on a hexagonal grid (0.6 cm spacing) requiring island blocks in a direction emanating from the source position, assumed 95 cm upstream of the foam board's downstream surface. Each hole's diameter was slightly less than the island block diameter required at that location, ensuring the island blocks fit snugly. Second, island blocks of the specified diameter, selected from the set of diameters in Figure 1, were manually inserted into each hole. Third, the foam board was milled to shape and pressed into a milled copper cutout^24^ defining the extent of the radiation field. Hilliard et al.^19^ illustrated PRIME devices fabricated using this method for head and postmastectomy chest wall patients.

### 2.2 Description of test PRIME devices

Six test PRIME devices were used in this study, each manufactured to collimate the field to 21 × 21 cm^2^ at isocenter. The devices contained an intensity modulated region approximately 8 × 8 cm^2^ centered on central axis that consisted of 247 island blocks arranged in a regular hexagonal grid (packing radius 0.6 cm). All island blocks in each PRIME device were of constant diameter, three being 0.158 cm and three being 0.352 cm, corresponding to IRF values of 0.937 and 0.688, respectively. For both diameters, one PRIME device was fabricated with a systematic error in that the axis of the island blocks incorrectly projected to a source 95 cm downstream of the block, such that the blocks were aligned convergently rather than divergently, that is, having a large systematic error that masked random errors. A second PRIME device was fabricated with axes of its island blocks correctly projecting to a source 95 cm upstream, but in which some island blocks were inserted with a considerable random error about the perfectly‐aligned axis that is, having negligible systematic error but large random errors. A third PRIME device was fabricated with proper divergence and using an improved method for manual insertion of the island blocks that decreased random error that is, having negligible systematic error and small random errors. All six of these PRIME devices passed optical imaging QA at the factory, that is, the upstream surfaces of the island blocks were of proper diameter and location was accurate (within 0.07 cm).

### 2.3 Acquisition of x‐ray radiographs

X‐ray images of PRIME devices were acquired using a GE AMX4 Plus mobile kV x‐ray unit (Model 270954G1, S/N 15931, GE Healthcare, Chicago, IL, USA). Maximum image contrast was selected experimentally by acquiring images at various existing clinical kVp and mAs settings (52 kVp, 2 mAs to 100 kVp, 20 mAs). As expected, the 52 kVp setting was selected due to providing the best image contrast. Further optimization was not explored due to the high contrast of tungsten being adequate for determining island block projections. A Vieworks VIVIX (Vieworks Co., Ltd., Anyang, South Korea) flat panel detector was used to acquire the x‐ray images with a pixel resolution of 0.014 cm. To minimize object blur, the PRIME devices were laid on the proximal surface of the flat panel detector.

The source to detector distance was set to 95 cm, replicating the position of the PRIME device in the electron applicator and closely reproducing the divergence of the therapeutic electron beam. The central axis of the PRIME device was then aligned with the central axis of the graticule of the x‐ray unit and positioned such that the +*X* and +*Y* directions of the PRIME device (cutout coordinates) were closely aligned with the columns and rows of the image, respectively. Image exposure times were controlled by the unit, which operated using default settings from the manufacturer. Images were automatically sent from the flat panel detector to the accompanying VXvue (Vieworks) workstation, where they were exported for external processing via DICOM transfer.

### 2.4 Image processing

Pixel data were read from the DICOM x‐ray image files using an in‐house Python (version 3.7) script. To segment the applicator cutout and island blocks in the image, linear contrast scaling was applied, then a threshold was selected to generate a binary mask. Regions in the binary mask were classified as island blocks, the cutout, or other objects (e.g., radiopaque marker) based on the pixel area of each region.

A transformation matrix mapping pixel location in the image to the cutout coordinates was determined from the rotation angle and centroid of the minimum‐area bounding rectangle^25^ that encompassed the outer border of the cutout. This placed the imaged pixels and the planned positions of the centers of the island blocks in the same coordinate system for further calculations.

### 2.5 Determination of island block misalignment

The island blocks are right cylinders whose axes should be aligned with the divergence of the beam, and an island block that is correctly aligned will have a projection (shadow) onto the imaging plane that can be modeled circular. Misalignment of the island block axis with the beam divergence will result in a projection that can be modeled as two semi‐ellipses separated by some distance, with the eccentricity of the ellipses and distance between ellipses dependent on the angle of misalignment. Based on these principles, a method was developed to quantify island block misalignment using a geometrical model and an input x‐ray image.

**FIGURE 2:**
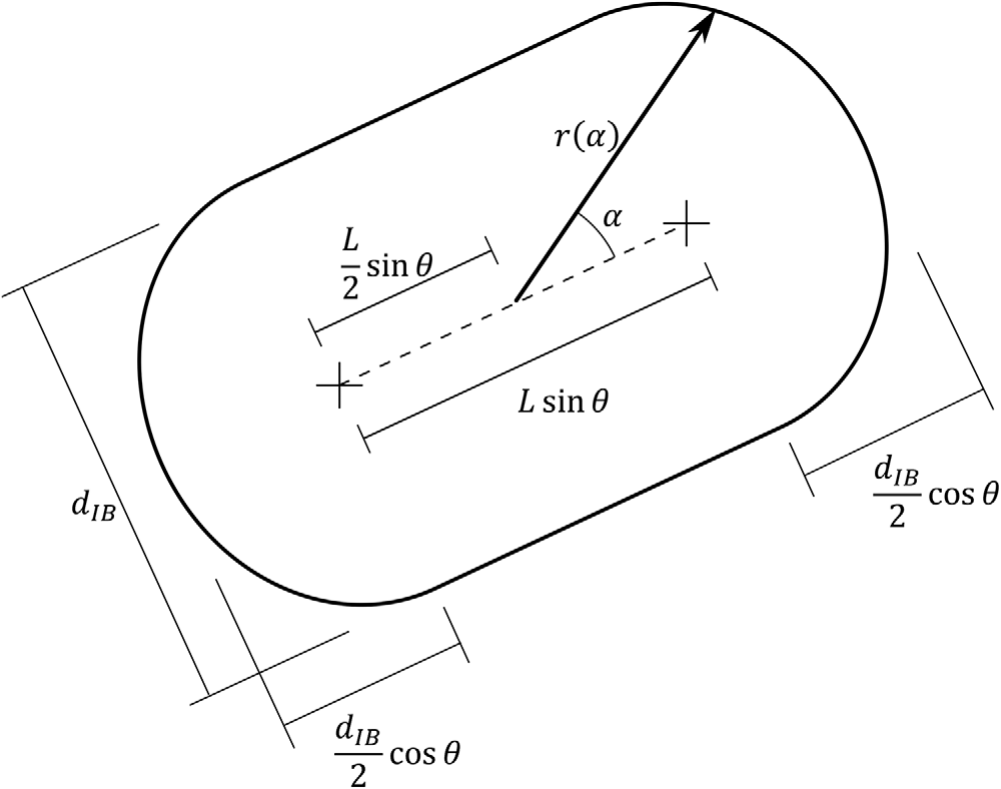
Shape of outline of misaligned island block x‐ray image projected onto image plane. The “racetrack” shape has a semi‐ellipse on each end (major axis length *d_IB_* and minor axis length *d_IB_* cos*θ* where *d_IB_* is the island block diameter and *θ* is the polar angle of misalignment, connected by a rectangle of length *L*sin*θ*, where *L* is the length of the island block (0.6 cm). In polar coordinates *r(α)* is given by Equation 2. The angle *α* is determined relative to the line (dashed) passing through the semi‐ellipse centers (“+” markers).

The geometrical model for the projected image of a misaligned island block is two semi‐ellipses connected by a rectangle (*cf*. Figure 2). The ellipses forming the ‘ends’ of the island block projection will have a major axis length *d_IB_* and minor axis length of *d_IB_* cos*θ*, where *d_IB_* is the diameter of the island block and *θ* is the angle of misalignment (tilt) relative to the island block's diverging ray line, restricted to the range [0, π/2], although in practice *θ* « π/2 . The separation distance of the centers of the ellipses is equal to *L*sin*θ*, where *L* is the length of the island block (0.6 cm). The perimeter (racetrack) of the island block projection can be expressed in polar coordinates, with α being the polar angle defined over the range [0, 2π) and *r(α)* being the distance of the perimeter from the center of the projection. The projection perimeter is provided as a piecewise function, which handles separately the elliptical and linear sections. The elliptical sections (“turns” of the racetrack) are defined for α within $\frac{\pi }{2}-\mathrm{atan}\left(\frac{L\sin\theta }{d_{IB}}\right)$ radians of the nearest integer multiple of π,

(1) $$\begin{array}{ccc} r_{ellipses}\left(\alpha \right) & = & \frac{1}{2}\{L\left|\cos\alpha \right|\tan\theta \sec\theta \\ & & +\left[L^{2}\cos^{2}\alpha \tan^{2}\theta \sec^{2}\theta \right.\\ & & -\left.{\left.(1+\cos^{2}\alpha \tan^{2}\theta )\left(L^{2}\tan^{2}\theta -d_{IB}^{2}\right)\right]}^{\frac{1}{2}}\right\}\\ & & \times \;{\left(\cos^{2}\alpha \tan^{2}\theta +1\right)}^{-1}. \end{array}$$

The complete piecewise function is expressed as

(2) $$r\left(\alpha \right)=\left\{\begin{array}{cc} r_{ellipses}\left(\alpha \right)\;\; & \;\left|\alpha -\mathrm{Round}\left(\frac{\alpha }{\pi }\right)\pi \right|\leq \frac{\pi }{2}-\mathrm{atan}\left(\frac{L\sin\theta }{d_{IB}}\right)\\ \frac{d_{IB}}{2\left|\sin\left(\alpha \right)\right|} & \;\mathrm{Otherwise} \end{array},\right.$$

where Round is the rounding function to the nearest integer.

The polar angle α is relative to the line passing through the major axis of the island block projection, which passes through the minor axes of the semi‐ellipses at each end. This model ignores perspective zoom, i.e., that one of the sides of the island block is closer to the source and projects slightly larger on the imager. However, given that the distance from the source to the island blocks (95 cm) is much larger than the length of the island blocks (0.6 cm), the upstream island block surface will appear on the imager only 0.6% wider than the downstream surface. For a 0.352 cm diameter island block, a pixel resolution of 0.002 cm is required to observe a 1‐pixel difference between the projected upstream and downstream diameters, which is not available on the imaging system used.

This model also assumes that each island block, if perfectly aligned, would project a circle onto the imager. This assumption is imperfect, as the imaging plane cuts through each island block projection at an angle equal to the polar angle of divergence *θ_i_* of the ray‐line drawn from the source and passing through that island block (index *i*). The resultant, theoretical projection is an ellipse with major axis $d_{IB}/\cos\theta_{i}$ and minor axis *d_IB_* where the major axis is aligned with the projection of the diverging ray line onto the imager. Hence, the true projected area of the island block onto the imager is larger than the actual cross‐sectional island block area by a factor of $1/\cos\theta_{i}$. However, this is a small effect: for an island block located at (*x_i_, y_i_*) = (5 cm, 5 cm), the error in cross‐sectional area determination would be only 0.28%. Such error corresponds to a 0.1% absolute reduction in IRF for a 0.352 cm diameter island block, thus we considered this error negligible in comparison to much larger errors that could be caused by island block misalignment.

**FIGURE 3:**
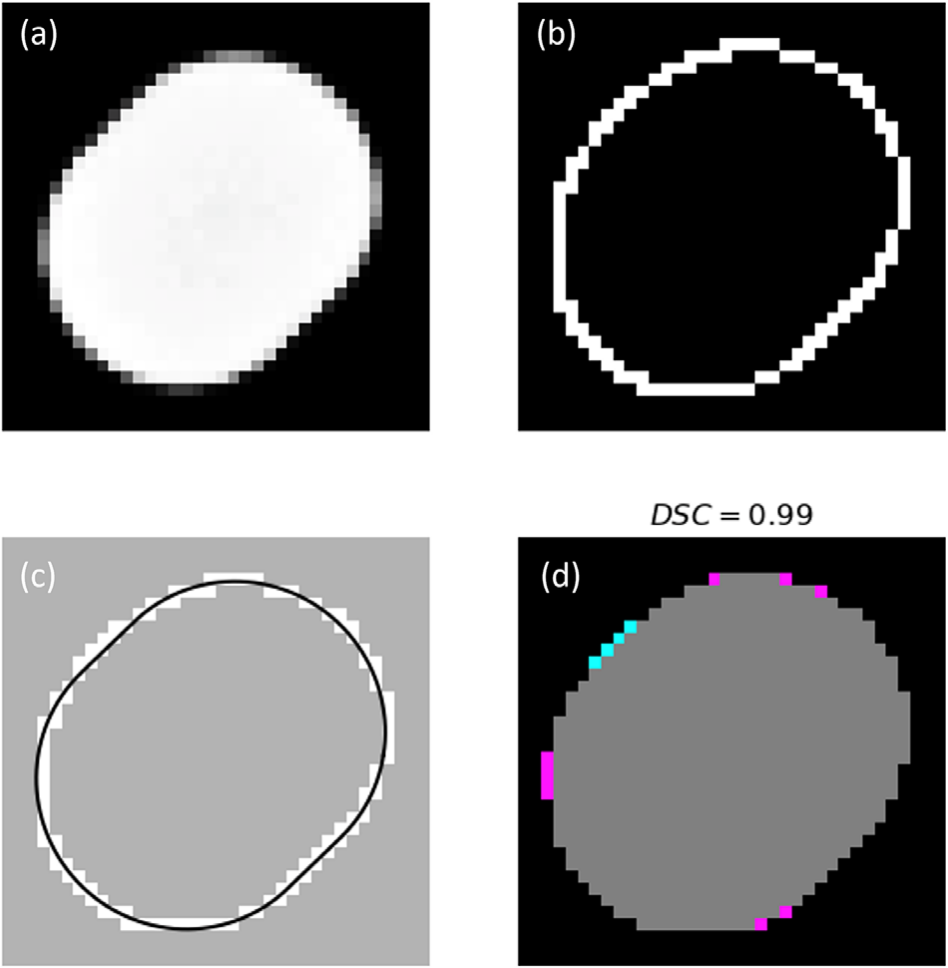
Fitting procedure to determine the amount and direction of misalignment for island blocks in PRIME devices. (a) Cropped grayscale pixel data from input x‐ray image. (b) Edge pixels (white) as determined from Canny edge detection. (c) Best fit curve (black) to center points of edge pixels. (d) Dice similarity coefficient (DSC) calculation as measure of fitting quality, where the gray pixels fall within both the input binary mask and the fit line, cyan pixels are present in the input binary mask but do not fall within the fit line, and magenta pixels fall within the fit line but not in the input binary mask.

The geometrical model described above was fit to the edge coordinates (perimeter) of each island block projection using a nonlinear least squares routine.^26^ The fitting procedure was comprised of the following six steps: (1) a binary mask was generated of each island block by thresholding the input x‐ray image, (2) the edge coordinates in the binary mask were determined using Canny edge detection, (3) the Canny detector produced a second binary mask where each pixel was assigned a value of 1 for having been detected as an edge, and 0 otherwise, (4) the edge coordinates were found as the integer pixel coordinates of pixels having a value of 1, (5) the edge coordinates were converted from the Cartesian coordinate system (row and column location in the image) to polar coordinates using the centroid of the island block's binary mask, and (6) fitting parameter *d_IB_* and *θ* best fitting the edge coordinates were determined. As a check of the fitting quality, the Dice similarity coefficient (DSC)^27^ comparing the fitting result to the input data was computed for each island block,

(3) $$DSC=\frac{2\left|B_{input}\cap B_{fit}\right|}{\left|B_{input}\right|+\left|B_{fit}\right|}\;,$$

where *B*_input_ and *B*_fit_ are the binary masks representing the input data and best fitting closed curve, respectively. *DSC* is a measure of the overlap between *B*_input_ and *B*_fit_, where a value of 1 corresponds to complete overlap and a value of 0 corresponds to no overlap. Figure 3 illustrates the fitting procedure for a sample 0.352 cm diameter island block.

### 2.6 Determination of island block location, diameter, and angle

The resulting fit yields the block diameter (*d_IB_*) and location of the centers of the upstream and downstream block surfaces, from which the polar angle of misalignment (*θ*) can be determined. Based on comparisons of optical and x‐ray QA images taken of the PRIME devices, results showed that the location of the center of the upstream island block surface (visible in the optical image) consistently agreed with the center of semi‐elliptical end of the island block in the x‐ray image nearest to its expected position. A vector from the center of the upstream ellipse to the center of downstream ellipse was then defined. The azimuthal angle of misorientation, *ϕ*, was measured as the angle of that vector with respect to the vector drawn from the origin (central axis) to the center of the upstream ellipse, counterclockwise in the imaging plane.

### 2.7 Geometry of island block misalignment

**FIGURE 4:**
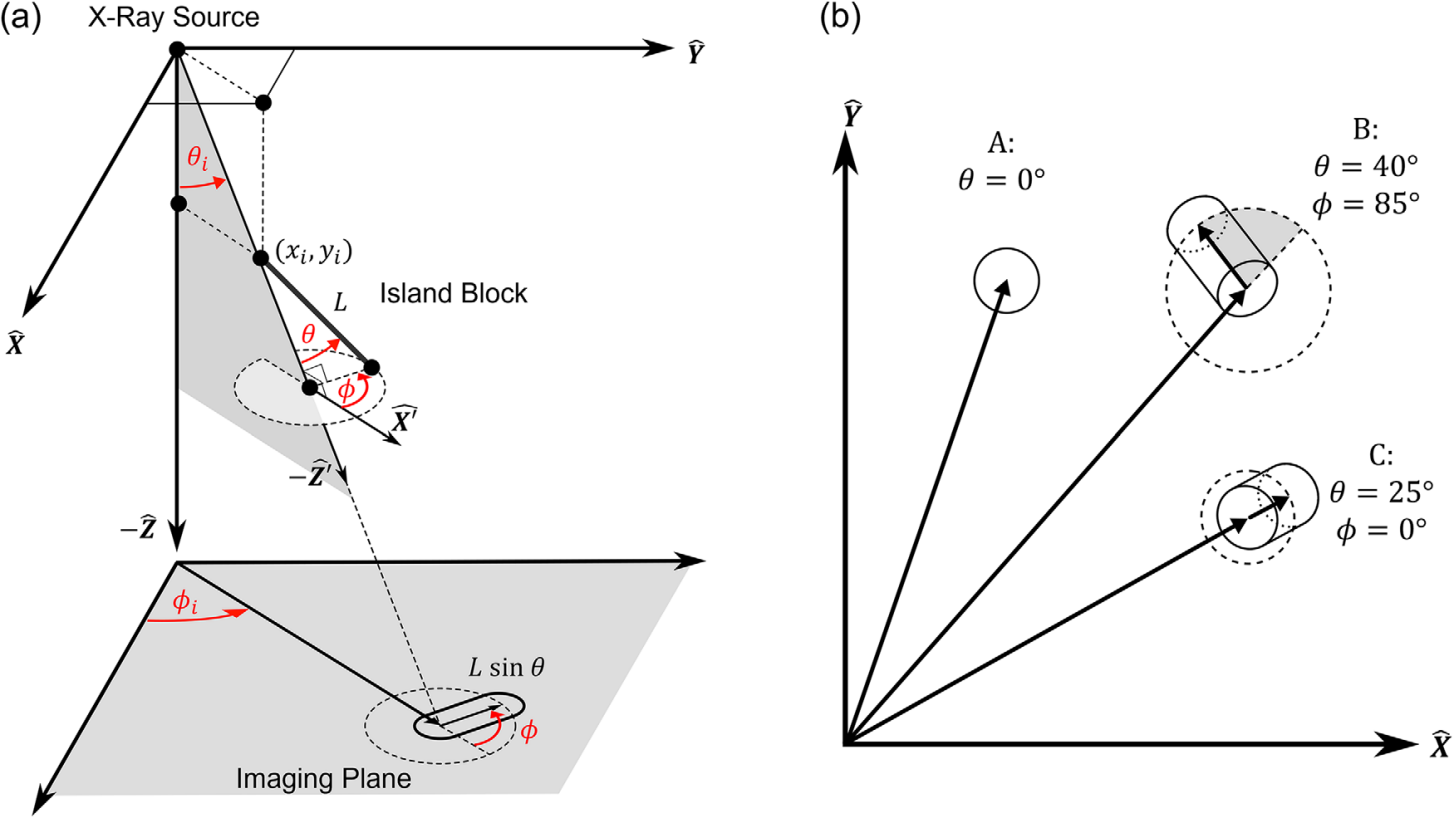
Geometry of island block misalignment. (a) Projection (not to scale) of island block (*Z* = −93.5 cm) from x‐ray source (*Z* = 0 cm) onto the imaging plane (*Z* = −95 cm). The *X* and *Y* axes of the cutout are displayed, with the −*Z* axis pointing toward isocenter, and the source located at the origin. The cylindrical axis of a misaligned island block (angles *θ* and *ϕ*, length *L*) is shown, along with the diverging ray passing through the island block's upstream ellipse center (*x_i_, y_i_*) and specified by angles *θ_i_* and *ϕ_i_*. Also included are the *Xˊ* and −*Zˊ* axes of the island block's coordinate system, in which *θ* and *ϕ* are defined. Finally, the racetrack shape of the island block projection onto the imaging plane is illustrated under the small angle approximation (*θ_i_* ≈ 0). (b) Example projections of island blocks of various orientations (*θ*, *ϕ*) in the imaging plane are shown. Case A illustrates perfect alignment. Case B illustrates an island block misoriented with large *θ* and *ϕ*, representative of the island block in (a). Case C illustrates an island block misaligned with respect to its diverging ray (*θ* ≠ 0) while having *ϕ* = 0. Vectors extending from the origin (central axis) to the center of the upstream projected ellipses (drawn as closed ellipses) make angles *ϕ_i_* counterclockwise with respect to the *X* axis. Nonzero *ϕ* (shaded region) is illustrated in case *B*. The length of each vector from the upstream to downstream projected ellipses is given by *L* sin *θ* as shown in (a).

Figure 4a illustrates the general case of island block misalignment. In Figure 4a, *θ* and *ϕ* are shown as they relate to *θ_i_* and *ϕ_i_*, where *ϕ_i_* is the azimuthal angle of divergence measured counterclockwise from the +*X* axis toward the +*Y* axis. The azimuthal angle of island block misorientation as measured from the positive *X*‐axis can be calculated using *ϕ* + *ϕ_i_*. Also illustrated are the rotated Cartesian coordinates (*Xˊ, Yˊ, Zˊ*), of the island block, in which *θ* and *ϕ* are defined in the general case. In these rotated coordinates, the −*Zˊ* axis is aligned with the diverging ray, pointing away from the source. The *Xˊ* axis lies in the *ZZˊ* plane, is normal to the *Zˊ* axis and points away from the *Z* axis. While not pictured, the *Yˊ* axis lies normal to the *ZZˊ* plane (shaded region), and points counterclockwise from the source perspective ($\hat{Y^{\prime }}=\hat{Z^{\prime }}\times \hat{X^{\prime }}$). In the general case, *θ* is defined as the polar angle of the island block axis with respect to the ‐*Zˊ* axis, and *ϕ* is defined as the azimuthal angle (counterclockwise) of the island block axis with respect to the *Xˊ* axis toward the *Yˊ* axis. In the special case of an island block located at central axis (*θ_i_* = 0), the island block coordinates are aligned with the cutout coordinates.

Due to the small *θ_i_* of the island blocks studied in this work (cos *θ_i_* ≈ 1), transformation from the imaging plane to these rotated coordinates was not necessary to determine *θ* and *ϕ* as the imaging (*XY*) and *XˊYˊ* planes were nearly parallel. However, such transformation might be necessary for island blocks near the edge of large fields (e.g., 25 × 25 cm^2^), where the small angle approximation is less applicable. Further illustration of angles *θ* and *ϕ* is provided in Figure 4b, where these angles are diagrammed for three possible projections. Hence, this fitting procedure yields the measured position, angular deviation (*θ, ϕ*) from the diverging ray line from the source (*x_i_, y_i_*), and diameter (*d_IB_*) of each island block for QA analysis.

### 2.8 Intensity maps

To assess the impact of island block positioning and orientation errors on the underlying dose distribution, intensity maps beneath each PRIME device were calculated using a pencil beam algorithm (PBA) for both perfect positioning and that determined from the x‐ray image. For the former PBA calculation, the projected circular cross section of each island block was assumed a square pencil beam of equal area whose edges paralleled the x and y axes. For the latter PBA calculation, first the nearest nominal diameter *d_nom_* to the fitted diameter *d_IB_* was selected from a finite set of five diameters used in construction of PRIME device, which are shown in Figure 1. To reduce the sensitivity of the calculated intensity map to variance in image contrast, *d_nom_* was used in the PBA calculation rather than *d_IB_*. Next, the projected racetrack image of each island block was modelled in the PBA as a rectangular pencil beam of equal area, with its long axis parallel to the racetrack's major axis. The major (*d_nom_* cos *θ + L* sin *θ)* and minor (*d_nom_*) axes of the racetrack were equally scaled to provide the width and length of the rectangle. The center of each pencil beam was that of the circular and racetrack images, respectively. A 16 MeV beam was selected because higher electron beam energies are more typical of BECT utilization in the clinic, and their intensity maps should be more sensitive to island block position or alignment errors.

The underlying map of the intensity distributions were calculated using the Hogstrom pencil beam algorithm^28^ following the method described by Chambers.^22^, ^29^ In this method, the intensity distribution for the pencil beam modeling the shape of each island block was subtracted from the pencil beam dose distribution computed for the field without island blocks. All pencil beams originated at the applicator (95 cm from the source) with initial projected angular spread *σ_θx_* and were propagated to a plane parallel to the surface of a water phantom (103 cm SSD, air gap = 8 cm). Values for *σ_θx_* and most probable energy at surface, *E_p,0_*, were taken from clinical commissioning data for the Elekta Infinity linear accelerator at Mary Bird Perkins Cancer Center, which were determined following Hogstrom et al.^30^ For the 16 MeV beam used for the calculations, *E_p,0_ =* 15.8 MeV and *σ_θx_* = 0.038 radians. To account for electron scattering in the machinable foam in which the island blocks were embedded, *σ_θx_* was increased by 50%, following Hilliard et al.^19^ To account for energy loss in the foam, *E_p,0_* was decreased by 0.25 MeV. All intensity maps were computed at a depth of 2 cm, an estimate of average thickness of overlying bolus and a sufficiently shallow depth to be sensitive to the impact of fabrication errors on the underlying dose distribution. All calculations were normalized such that 100% equaled central axis intensity of the collimated beam with foam, but without island blocks. For each PRIME device, a comparison of the intensity maps calculated beneath the designed and fitted island block projections was performed.

## 3 RESULTS

### 3.1 X‐ray images for PRIME devices

**FIGURE 5:**
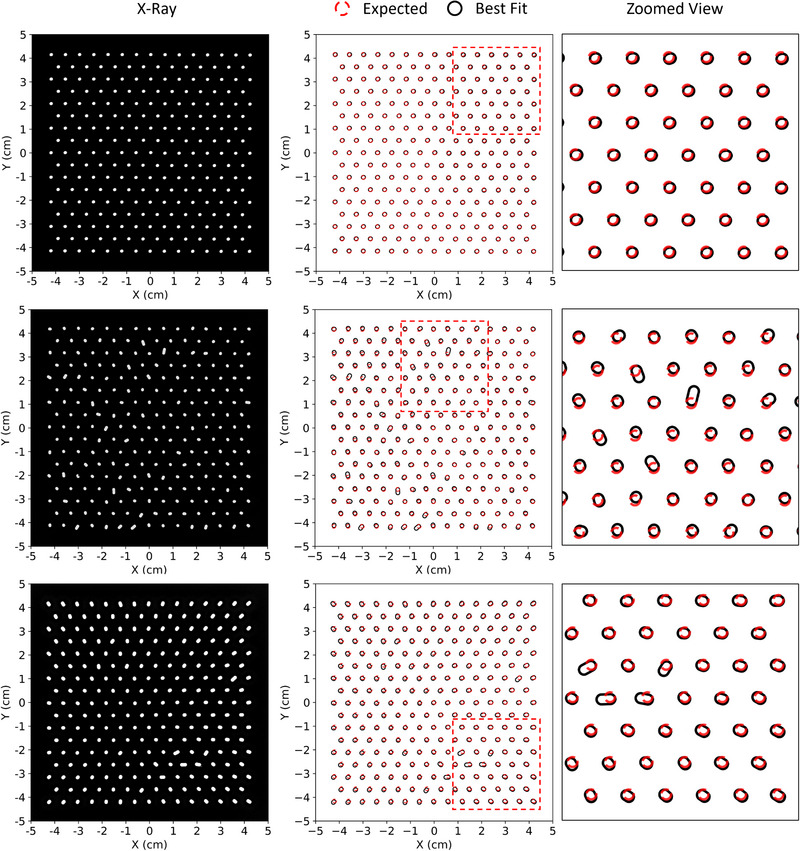
Comparison of x‐ray images of PRIME devices having 0.158 cm diameter island blocks. The top row shows results for the device with negligible systematic error and small random errors. The middle row shows results for the device with negligible systematic error but large random errors. The bottom row shows results for the device with large systematic error and random errors which have been masked by the systematic error. Left column shows the x‐ray images with the shadows of the island blocks outlined by white racetracks. Center column compares those racetracks (black) with the designed circular cross sections (red) on the hexagonal grid (0.6 cm spacing). Right column provides zoomed view (approx. 3.8 × 3.8 cm^2^ area) of selected comparisons between racetracks and designed circular cross sections.

**FIGURE 6:**
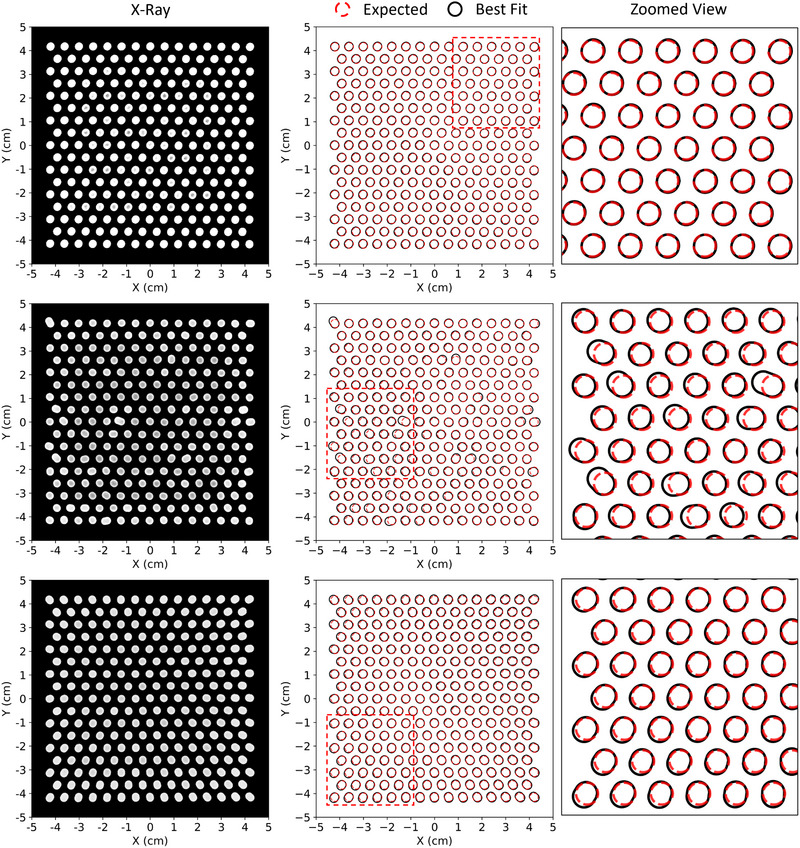
Comparison of x‐ray images of PRIME devices having 0.352 cm diameter island blocks. The top row shows results for the device with negligible systematic error and small random errors. The middle row shows results for the device with negligible systematic error but large random errors. The bottom row shows results for the device with large systematic error and random errors which have been masked by the systematic error. Left column shows the x‐ray images with the shadows of the island blocks outlined by white racetracks. Center column compares those racetracks (black) with the designed circular cross sections (red) on the hexagonal grid (0.6 cm spacing). Right column provides zoomed view (approx. 3.8 × 3.8 cm^2^ area) of selected comparisons between racetracks and designed circular cross sections.

X‐ray images for the test PRIME devices (intensity modulators) are shown for 0.158 cm diameter (IRF = 0.938) and 0.352 cm diameter (IRF = 0.689) island blocks in the left column of Figures 5 and 6, respectively. Plotted adjacently in the center column are comparisons of outlines of perfectly positioned and oriented island blocks (red circles) along with racetrack outlines determined from fits to the measured x‐ray image (black racetracks). A zoomed view of the comparisons is provided in the right column. For all devices, Dice coefficients (*DSC*) calculated for each fit were greater than 0.966, with 97% of fits having DSC values exceeding 0.98. The positions of the center of the racetrack's semi‐ellipse closest to the planned position of the center of the circles agree within 0.03, 0.05, and 0.07 cm for the devices with negligible systematic error and small random error, negligible systematic error and large random error, and large systematic error with masked random error, respectively for the 0.158 cm diameter island blocks. Similar results show agreement within 0.03, 0.05, and 0.05 cm for the 0.352 cm diameter island blocks. These results confirm that the island blocks were properly positioned using insertion guide holes.

**FIGURE 7:**
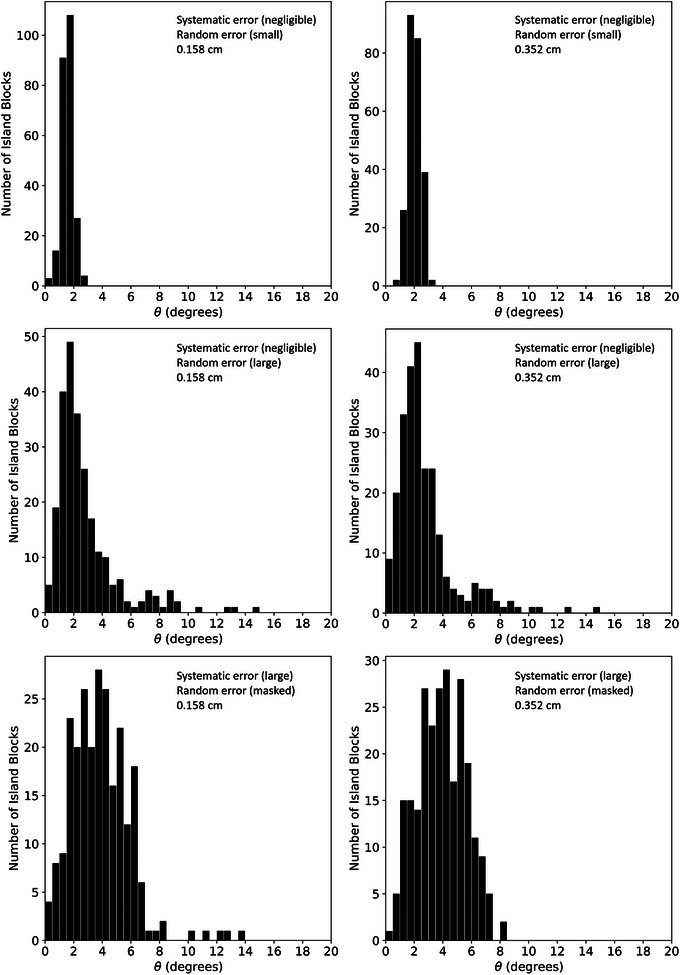
Distribution of polar angle of misalignment (*θ*) of island block axes from beam divergence. A legend is provided in the top right corner of each histogram indicating the nature of errors and the island block diameter for that device.

Comparison of the planned and measured island block outlines for PRIME devices having negligible systematic errors and small random errors with 0.158 cm and 0.352 cm diameter island blocks show very close agreement, indicating the island blocks are not only well positioned, but also their axes are closely aligned with diverging rays from the source. Using the described methodology, the racetrack dimensions provided values of *θ* which are histogrammed for all devices in Figure 7. As the figure shows, for devices with 0.158 cm and 0.352 cm diameter island blocks, *θ* values are less than 2.7° and 3.2°, respectively, for all island blocks within the two devices with negligible systematic error and small random error.

Comparison of the planned and measured island block outlines for the PRIME devices with 0.158 cm and 0.352 cm diameter island blocks and negligible systematic error but large random errors showed greater random differences, indicating the island block axes deviate from the diverging rays more than do the test devices with small random error. This is evident visually by the location, size, and orientation of the projected racetracks appearing to vary randomly. Again, using the racetrack shape, values of *θ* were determined and histogrammed in Figure 7, which illustrates *θ* being as great as 8.5° for several island blocks and a few outliers as great as 15.0° for the two devices with negligible systematic error but large random errors.

Comparison of the planned and measured island block outlines for the PRIME devices with 0.158 cm and 0.352 cm diameter island blocks with large systematic errors and masked random errors showed significantly broader *θ* distributions than the case where random errors were large but systematic errors were negligible. Also, the x‐ray images, particularly for the 0.158 cm island blocks, showed elongation of the racetrack images, which tended to point away from central beam axis and increased with distance from central beam axis. This is explained by the island blocks axes mistakenly being oriented to be converging to a source 95 cm downstream rather than diverging from a source 95 cm upstream. Again, using the racetrack shape, *θ* was determined and histogrammed in Figure 7. The resulting *θ* distributions for both diameters were significantly broader than those with random error only, with most values being within 7.5°, but a few outliers being just under 14.0° for the 0.158 cm diameter device.

### 3.2 Impact of island block alignment on intensity map

**FIGURE 8:**
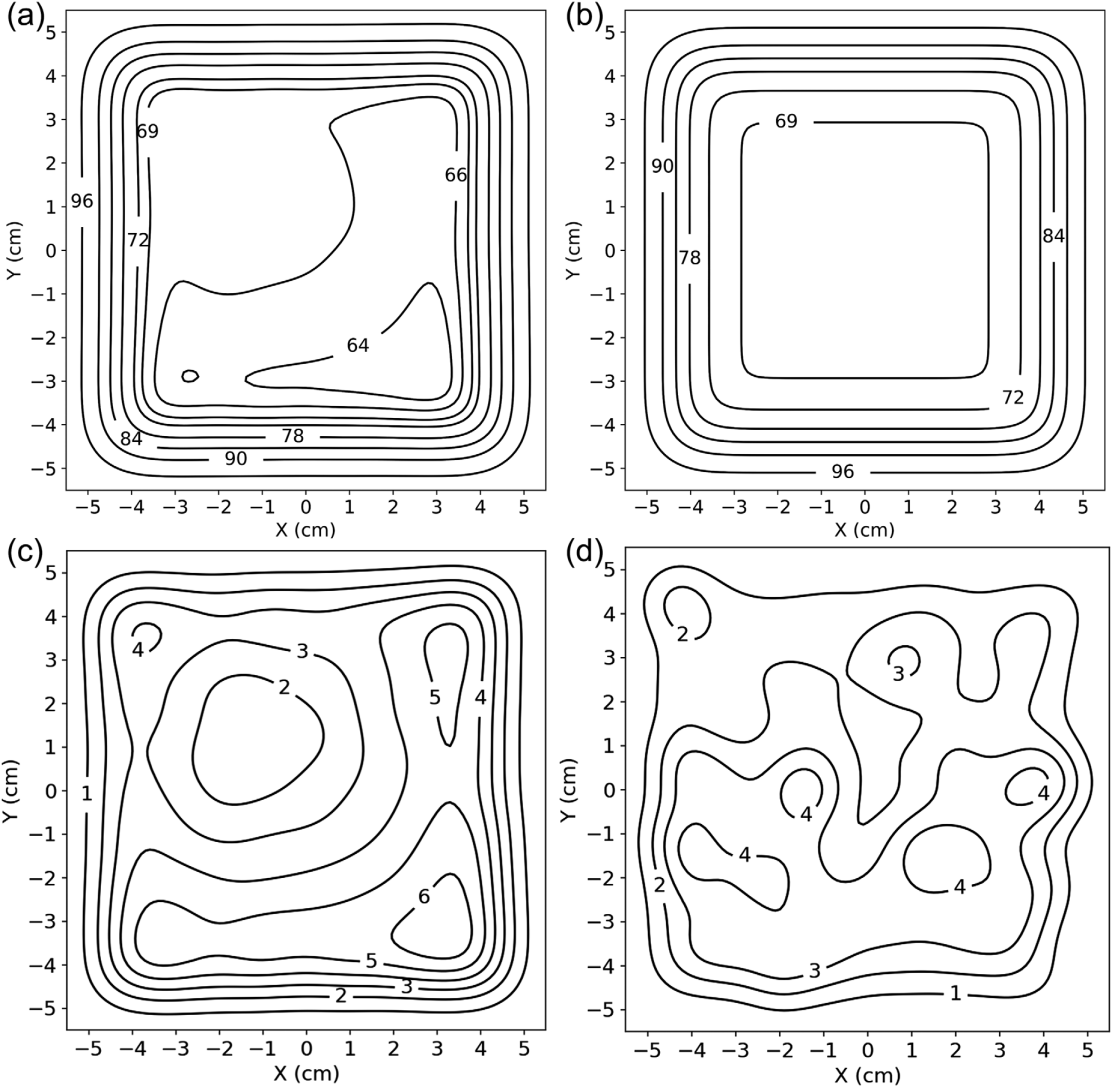
Comparison of PRIME device intensity maps calculated for planned and measured island block positions as compared in Figure 6 (PRIME devices with 0.352 cm diameter island blocks). (a) shows the intensity distribution calculated using measured racetrack projections of island blocks for the device with large systematic error. (b) shows the intensity distribution calculated using planned circular projections (proper locations and angulations). (c) shows calculated differences from planned intensity distributions (planned circular projections less measured racetrack projections) for PRIME device with systematic error. (d) shows calculated differences from planned intensity distributions for PRIME device with negligible systematic error but large random errors. Isointensity lines are shown for 10 × 10 cm^2^ region underlying island blocks; 100% equals central axis intensity with no island blocks present. All calculations were performed at 2 cm depth in water with 103 cm SSD.

The impact of errors in island block alignment was assessed by computing and comparing the underlying intensity maps for perfectly positioned (theoretical) and factory aligned (measured) island blocks. Figure 8 displays the results of these calculated intensity maps, showing the results for the PRIME device with 0.352 cm diameter island blocks with large systematic error and masked random error and with no error (theoretical) in Figure 8a,b, respectively. The difference between these intensity maps (theoretical minus measured) is shown in Figure 8c. To illustrate the effect of the nature of alignment errors, for example, systematic or random, on the underlying intensity map, the difference map for the PRIME device with 0.352 cm island blocks and negligible systematic error but large random errors is shown in Figure 8d.

**FIGURE 9:**
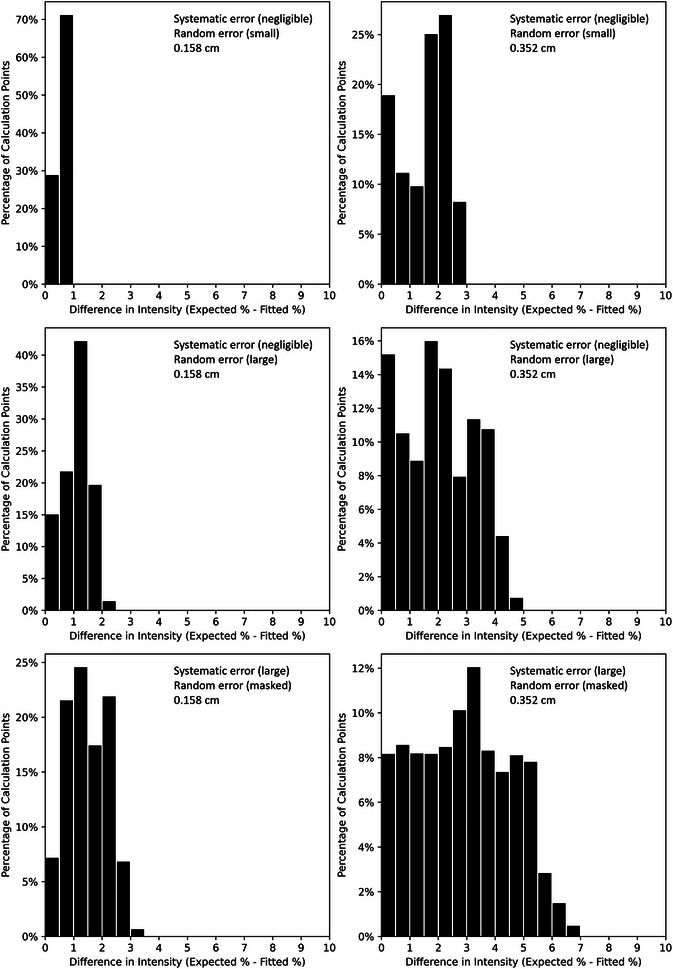
Histograms of differences in calculated intensity distributions (planned circular projections less measured racetrack projections) for PRIME devices. Calculations were performed using intensity distributions as exemplified in Figure 8. The histograms were limited to the calculated region inside the penumbra (calculations at 2‐cm depth in 103 cm SSD water phantom; area having <99% was histogrammed; modulated region ≈ 8 × 8 cm^2^). A legend in the top right corner of each histogram indicates the nature of errors and the island block diameter for that device.

These results are further analyzed in Figure 9, which shows histogram comparisons of calculated dose differences for all six test PRIME devices. For both the 0.158 and 0.352 cm diameter devices, calculated intensity maps derived from measured block distributions were compared with calculations of planned island block distributions. Because the island block cross section only increases with misalignment, the underlying intensity maps of the measured block distributions show only decreases with respect to the theoretical intensity maps. This increase in magnitude of intensity modulation is reflected in the histogram as positive differences.

As illustrated in Figure 9, the maximum (95^th^ percentile) intensity differences for PRIME devices with negligible systematic error and small random error with 0.158 cm and 0.352 cm diameters were 1.0% (0.9%) and 2.8% (2.6%), respectively. Greater intensity differences were observed for the PRIME devices with large random errors, as the maximum (95^th^ percentile) calculated intensity differences were 2.2% (1.9%) and 4.8% (4.0%), respectively. As was observed with the *θ* distributions for the devices with large systematic error, the distributions of intensity differences for those devices were broader than those with negligible systematic error but large random errors. Differences in intensity were more severe overall for the systematic error case, with maximum (95^th^ percentile) calculated intensity differences being 3.2% (2.6%) and 6.7% (5.5%), respectively.

## 4 DISCUSSION AND CONCLUSIONS

It was concluded that this work provides a viable, economical method for factory QA of PRIME devices (intensity modulators) using x‐ray radiography, which provides each island block's position, diameter, and angular orientation, all of which are required to assess the effect on the underlying intensity distribution. The method is an improvement over current optical quality assurance in that the latter is unable to evaluate angular orientation, which substantially impacts the resulting intensity distribution. Random errors in angular orientation are believed largely due to the current fabrication method of manual insertion of the high‐density tungsten island blocks (19.3 g·cm^−3^) into guide holes in low‐density machinable foam (0.096 g·cm^−3^).

Results showed that x‐ray radiographs can be used to determine errors in island block positioning, which can be random or systematic, allowing for informed quality evaluation of the fabrication process. Also, those radiographs provide data that allow calculation of underlying intensity maps to compare with those calculated for perfectly aligned island blocks. Although the two pencil beam calculations have small errors due to their ignoring scatter into or out of the sides of the blocks,^31^ their dose differences should be adequate for QA purposes. This is because the fluence of electrons scattered by the island blocks is small compared to the fluence removed by the island blocks.

**FIGURE 10:**
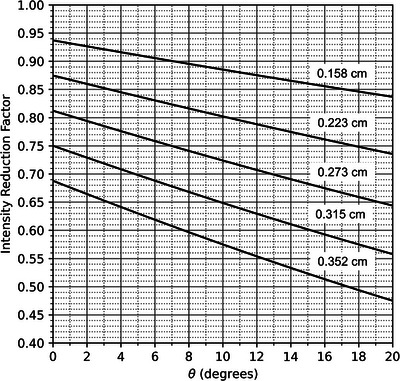
Plot of intensity reduction factor (IRF) versus *θ*, the tilt of the island block's axis from perfect alignment. Each line's island block diameter is demarcated. The corresponding IRF values are for placing the island blocks on a hexagonal grid (0.6 cm spacing). *θ* = 0° represents perfect alignment of the island block's axis with the beam's divergence. Misalignment of 2° can reduce the intensity by as much as 2.5% for the test PRIME devices investigated.

Also, although *θ* distributions were similar for the two island block diameters, dose difference distributions were significantly broader for the 0.352 cm compared to the 0.158 cm diameter island blocks. Hence, as illustrated in Figure 10, the broader diameter island blocks (lower IRF) require greater accuracy for equal dose differences.

The Canny edge detection method utilized in this study was found to be sufficiently accurate for determination of edge coordinates of the island blocks in the x‐ray images. Although other gradient‐based edge detection methods such as Sobel edge detection could provide increased accuracy, more accurate algorithms were deemed unnecessary due to the set of island block diameters being spaced greater than small errors in edge detection algorithms.

Uncertainties in the measurement method and in device manufacturing were ignored due to the much larger impact of island block misalignment on the underlying fluence distribution. For each device (247 island blocks of constant diameter), the standard deviation of diameters determined from the fitting procedure was 0.004 cm, which is well within the smallest spacing between nominal island block diameters (0.037 cm between 0.315 and 0.352 cm island blocks). Uncertainty in positioning of island blocks during manufacturing could not be separated from the uncertainty in the x‐ray QA method to determine the island block positions. However, the upper limit on each of these uncertainties was estimated by finding the standard deviations in the x‐ and y‐ differences between the determined positions and nominal positions, which were both 0.013 cm. To further assess the uncertainties in the x‐ray QA method, the fabrication and utilization of a ‘standard’ PRIME device with highly accurate island block positioning and alignment could be useful.

The determination of threshold values for pass rates remains under investigation. It is recommended that the passing criteria be based on the calculated intensity map, not distributions of misalignment parameters. Based on the current data, a passing criterion of 100% of the area within the penumbra agreeing to within 3% intensity seems achievable and appropriate. A distance criterion is unlikely needed, as measurements are evaluated inside the penumbra where intensity modulation exists without large dose gradients. As currently recommended passing criteria were determined from a limited number of test PRIME devices in the present study, future passing criteria will need to be determined from a significantly large sample of patient PRIME devices once intensity modulated bolus electron conformal therapy (IM‐BECT)^19^ becomes clinically available.

## AUTHOR CONTRIBUTIONS

As submitting author, I attest that all coauthors and myself (1) contributed to drafting and/or editing the submitted manuscript, (2) reviewed and approved the final submitted manuscript, and (3) are accountable for the integrity of the material submitted. Each individual contributed in multiple ways to the material reported in this manuscript, and the primary contribution(s) from each author are: Andrew S. McGuffey was a graduate student who contributed to development of the x‐ray QA method, developed the software used to process and analyze the x‐ray images, assisted with x‐ray image acquisition, and shared manuscript writing. Garrett M. Pitcher supervised the graduate student, contributed to data analysis, and shared manuscript writing. Rebecca L. Guidry assisted in x‐ray image acquisition, including determination of optimal x‐ray imaging parameters, and provided access to the x‐ray imaging equipment used in this study. Kevin J. Erhart developed the factory construction process for the PRIME devices, provided the PRIME devices used in this study, and served as principal investigator on the SBIR grant funding this study. Kenneth R. Hogstrom conceived the concept of intensity modulated bolus electron conformal therapy (IM‐BECT), developed the scientific portion of the grant that funded this project, contributed to data analysis, and shared manuscript writing.

## CONFLICT OF INTEREST STATMENT

Kevin Erhart is an employee of .decimal, LLC and principal investigator of NIH Award Number 2R44CA199838‐02 for which Mary Bird Perkins Cancer Center has a subaward. The authors have no other relevant conflicts of interest to disclose.
